# Supplementary material for: Evidence of shared and distinct functional and structural brain signatures in schizophrenia and autism spectrum disorder
Source: Commun Biol. 2021 Sep 14;4:1073. doi: 10.1038/s42003-021-02592-2 (PMC8440519; doi:10.1038/s42003-021-02592-2)
Supplement: Supplementary file 2 — Supplementary Information [file 42003_2021_2592_MOESM2_ESM.pdf]

Supplementary materials

Table S1 Demographic information and head motion measures of fMRI data for each dataset

**BSNIP**		**SZ**	**HC**	**p-value**
**(419 subjects: 182 SZ, 237 HC)**	age: mean (std)	35.11 (12.03)	38.13 (12.59)	0.0136
	gender: male/female	56/126	137/100	3.724e-08
	transitions: mean (std)	0.1451 (0.1485)	0.1269 (0.1030)	0.1397
	rotations: mean (std)	0.1159 (0.1193)	0.1312 (0.1205)	0.1968
**COBRE**		**SZ**	**HC**	**p-value**
**(157 subjects: 68 SZ, 89 HC)**	age: mean (std)	37.74 (14.4714)	38.09 (11.6625)	0.8652
	gender: male/female	57/11	64/25	0.0785
	transitions: mean (std)	0.1984 (0.1202)	0.2184 (0.1464)	0.3624
	rotations: mean (std)	0.1769 (0.1208)	0.1891 (0.1177)	0.5258
**FBIRN**		**SZ**	**HC**	**p-value**
**(281 subjects: 137 SZ, 144 HC)**	age: mean (std)	39.02 (11.35)	37.15 (11.00)	0.1605
	gender: male /female	103/34	104/40	0.5733
	transitions: mean (std)	0.1790 (0.1269)	0.1921 (0.1465)	0.4239
	rotations: mean (std)	0.1982 (0.1592)	0.2132 (0.1551)	0.4223
**MPRC**		**SZ**	**HC**	**p-value**
**(388 subjects: 150 SZ, 238 HC)**	age: mean (std)	38.70 (14.0535)	40.24 (15.1675)	0.3186
	gender: male/female	98/52	94/144	7.158e-07
	transitions: mean (std)	0.1016 (0.1028)	0.0864 (0.0569)	0.0617
	rotations: mean (std)	0.0819 (0.0977)	0.0728 (0.0553)	0.2419
**ABIDEI**		**ASD**	**HC**	**p-value**
**(869 subjects: 398 ASD, 471 HC)**	age: mean (std)	17.75 (8.57)	17.62 (7.60)	0.8252
	gender: male/female	348/50	380/91	0.0071
	transitions: mean (std)	0.2018 (0.1314)	0.1798 (0.1148)	0.0084
	rotations: mean (std)	0.2103 (0.1377)	0.1925 (0.1237)	0.0459
**ABIDEII**		**ASD**	**HC**	**p-value**
**(866 subjects: 380 ASD, 486 HC)**	age: mean (std)	15.98(9.80)	14.79 (9.27)	0.0666
	gender: male/female)	327/53	342/144	4.682e-08
	transitions: mean (std)	0.1930 (0.1483)	0.1929 (0.1458)	0.9894
	rotations: mean (std)	0.2036 (0.1560)	0.1936 (0.1437)	0.3285

Note: Std denotes standard deviation. For each dataset, two-sample t-tests were used to examine the group differences in age, motion translations, and motion rotations. The motion translation measure of each subject was computed by averaging translation parameters across time points as well as x, y, and z axes. The motion rotation measure of each subject was computed by averaging rotation parameters across time points as well as pitch, yaw, and roll. A Chi-square test was performed for testing the gender difference.

Table S2 Demographic information of modulated sMRI data for each dataset

**BSNIP**		**SZ**	**HC**	**p-value**
**(374 subjects: 175 SZ, 199 HC)**	age: mean (std)	33.94 (11.69)	38.49 (12.63)	3.630e-04
	gender: male/female)	47/128	110/89	2.750e-08
**COBRE**		**SZ**	**HC**	**p-value**
**(140 subjects: 61 SZ, 79 HC)**	age: mean (std)	35.07 (13.50)	37.89 (11.44)	0.1840
	gender: male/female	54/7	57/22	0.0178
**FBIRN**		**SZ**	**HC**	**p-value**
**(294 subjects: 149 SZ, 145 HC)**	age: mean (std)	39.30 (11.23)	37.75 (11.36)	0.2420
	gender: male/female)	114/35	105/40	0.4205
**MPRC**		**SZ**	**HC**	**p-value**
**(301 subjects: 132 SZ, 169 HC)**	age: mean (std)	37.45 (14.11)	41.15 (14.26)	0.0255
	gender: male/female)	94/38	76/93	5.198e-06
**ABIDEI**		**ASD**	**HC**	**p-value**
**(1034 subjects: 500 ASD, 534 HC)**	age: mean (std)	17.28 (8.01)	17.55 (7.75)	0.5876
	gender: male/female	440/60	444/90	0.0268
**ABIDEII**		**ASD**	**HC**	**p-value**
**(1005 subjects: 470 ASD, 535 HC)**	age: mean (std)	14.90 (8.63)	15.24 (9.15)	0.5451
	gender: male/female	397/73	372/163	2.501e-08

Note: Std denotes standard deviation. For each dataset, two-sample t-tests were used to examine the group differences in age. A Chi-square test was performed for the gender.

Table S3 Demographic information of unmodulated sMRI data for each dataset

**BSNIP**		**SZ**	**HC**	**p-value**
**(411 subjects: 181 SZ, 230 HC)**	age: mean (std)	33.94 (11.84)	38.59 (12.54)	1.55e-04
	gender: male/female	51/130	131/99	5.503e-09
**COBRE**		**SZ**	**HC**	**p-value**
**(151 subjects: 67 SZ, 84 HC)**	age: mean (std)	35.58 (13.79)	37.83 (11.63)	0.2780
	gender: male/female	58/9	61/23	0.0372
**FBIRN**		**SZ**	**HC**	**p-value**
**(308 subjects: 157 SZ, 151 HC)**	age: mean (std)	39.11 (11.43)	37.74 (11.47)	0.2910
	gender: male/female	119/38	110/41	0.5536
**MPRC**		**SZ**	**HC**	**p-value**
**(349 subjects: 150 SZ, 199 HC)**	age: mean (std)	37.39 (13.87)	40.86 (14.36)	0.0239
	gender: male/female	102/48	84/115	1.749e-06
**ABIDEI**		**ASD**	**HC**	**p-value**
**(1077 subjects: 522 ASD, 555 HC)**	age: mean (std)	17.14 (8.15)	17.32 (7.72)	0.7176
	gender: male/female	458/64	461/94	0.0302
**ABIDEII**		**ASD**	**HC**	**p-value**
**(1078 subjects: 508 ASD, 570 HC)**	age: mean (std)	14.65 (8.69)	15.02 (9.20)	0.4991
	gender: male/female	432/76	395/175	1.034e-09

Note: Std denotes standard deviation. For each dataset, two-sample t-tests were used to examine the group differences in age. A Chi-square test was performed for the gender.

Table S4 Demographic information and head motion measures of fMRI data and demographic information of sMRI data from all datasets

		SZ	ASD	HC	p-value
**fMRI data from all datasets (2980 subjects: 1665 HCs, 537 SZs, and 778 ASDs)**	age: mean (std)	37.4 (12.9)	16.9 (9.2)	25.7 (15.5)	2.03e-141
	gender: male/female	314/223	675/103	1121/544	3.154e-32
	transitions: mean (std)	0.1484 (0.1321)	0.1975 (0.1399)	0.1659 (0.1283)	2.0e-11
	rotations: mean (std)	0.1351 (0.1337)	0.2070 (0.1469)	0.1686 (0.1330)	3.5e-20
**Modulated sMRI data from all datasets (3148 subjects: 1661 HCs, 517 SZs, and 970 ASDs)**		**SZ**	**ASD**	**HC**	**p-value**
	age: mean (std)	36.5 (12.6)	16.1 (8.4)	24.4 (14.9)	2.98e-165
	gender: male/female	309/208	837/133	1164/497	2.344e-31
**Unmodulated sMRI data from all datasets (3374 subjects: 1789 HCs, 555 SZs, and 1030 ASDs)**		**SZ**	**ASD**	**HC**	**p-value**
	age: mean (std)	36.5 (12.7)	15.9 (8.5)	24.6 (15.1)	2.79e-176
	gender: male/female	330/225	890/140	1242/547	7.838e-35

Note: Std denotes standard deviation. For each type of data (e.g. fMRI), analysis of variance (ANOVA) methods were used to examine the differences of HC, SZ, and ASD in the age, motion translations, and motion rotations. A Chi-square test was performed for the gender.

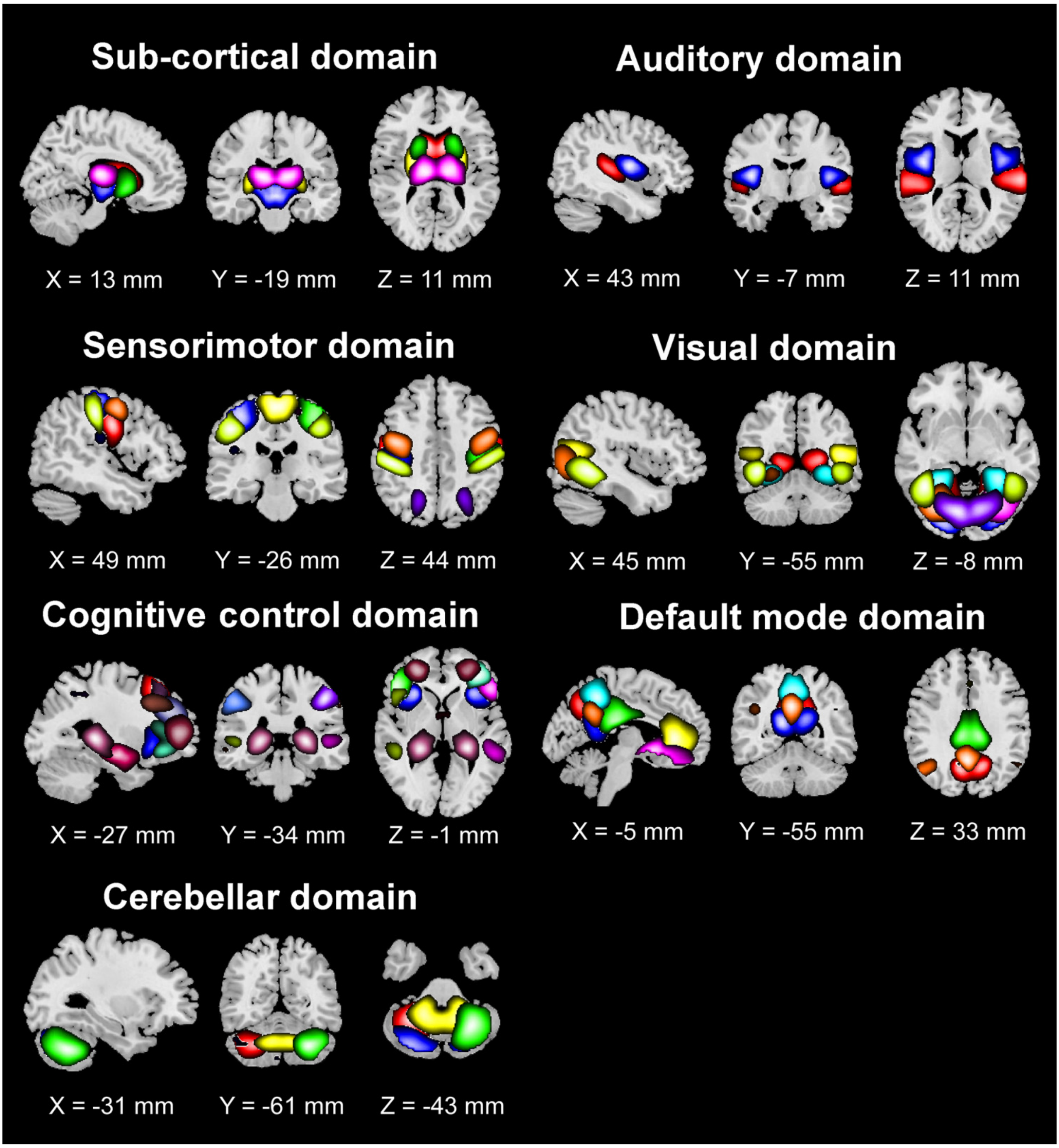

Fig. S1 The network templates used in the NeuroMark. These networks were assigned to seven functional domains including sub-cortical (SC: 5), auditory (AU: 2), sensorimotor (SM: 9), visual (VI: 9), cognitive control (CC: 17), default mode (DM: 7) and cerebellar (CB: 4) domains. Here, different networks in the same functional domain are displayed using different colors.

Table S5. Information of the network templates. For each network template, its functional domain, primary brain region, and peak coordinate are included. Here, each network template is represented by one independent component (IC). The IC ID is shown along with the brain region name.

**Primary regions in networks (IC ID)**	**X**	**Y**	**Z**	**Primary regions in networks (IC ID)**	**X**	**Y**	**Z**
**Sub-cortical domain (SC)**				**Cognitive control domain (CC)**			
Caudate (IC 69)	6.5	10.5	5.5	Inferior parietal lobule ([IPL], IC 68)	45.5	-61.5	43.5
Subthalamus/hypothalamus (IC 53)	-2.5	-13.5	-1.5	Insula (IC 33)	-30.5	22.5	-3.5
Putamen (IC 98)	-26.5	1.5	-0.5	Superior medial frontal gyrus ([SMFG], IC 43)	-0.5	50.5	29.5
Caudate (IC 99)	21.5	10.5	-3.5	Inferior frontal gyrus ([IFG], IC 70)	-48.5	34.5	-0.5
Thalamus (IC 45)	-12.5	-18.5	11.5	Right inferior frontal gyrus ([R IFG], IC 61)	53.5	22.5	13.5
**Auditory domain (AU)**				Middle frontal gyrus ([MiFG], IC 55)	-41.5	19.5	26.5
Superior temporal gyrus ([STG], IC 21)	62.5	-22.5	7.5	Inferior parietal lobule ([IPL], IC 63)	-53.5	-49.5	43.5
Middle temporal gyrus ([MTG], IC 56)	-42.5	-6.5	10.5	Left inferior parietal lobue ([R IPL], IC 79)	44.5	-34.5	46.5
**Sensorimotor domain (SM)**				Supplementary motor area ([SMA], IC 84)	-6.5	13.5	64.5
Postcentral gyrus ([PoCG], IC 3)	56.5	-4.5	28.5	Superior frontal gyrus ([SFG], IC 96)	-24.5	26.5	49.5
Left postcentral gyrus ([L PoCG], IC 9)	-38.5	-22.5	56.5	Middle frontal gyrus ([MiFG], IC 88)	30.5	41.5	28.5
Paracentral lobule ([ParaCL], IC 2)	0.5	-22.5	65.5	Hippocampus ([HiPP], IC 48)	23.5	-9.5	-16.5
Right postcentral gyrus ([R PoCG], IC 11)	38.5	-19.5	55.5	Left inferior parietal lobue ([L IPL], IC 81)	45.5	-61.5	43.5
Superior parietal lobule ([SPL], IC 27)	-18.5	-43.5	65.5	Middle cingulate cortex ([MCC], IC 37)	-15.5	20.5	37.5
Paracentral lobule ([ParaCL], IC 54)	-18.5	-9.5	56.5	Inferior frontal gyrus ([IFG], IC 67)	39.5	44.5	-0.5
Precentral gyrus ([PreCG], IC 66)	-42.5	-7.5	46.5	Middle frontal gyrus ([MiFG], IC 38)	-26.5	47.5	5.5
Superior parietal lobule ([SPL], IC 80)	20.5	-63.5	58.5	Hippocampus ([HiPP], IC 83)	-24.5	-36.5	1.5
Postcentral gyrus ([PoCG], IC 72)	-47.5	-27.5	43.5	**Default mode domain (DM)**			
**Visual domain (VI)**				Precuneus (IC 32)	-8.5	-66.5	35.5
Calcarine gyrus ([CalcarineG], IC 16)	-12.5	-66.5	8.5	Precuneus (IC 40)	-12.5	-54.5	14.5
Middle occipital gyrus ([MOG], IC 5)	-23.5	-93.5	-0.5	Anterior cingulate cortex ([ACC], IC 23)	-2.5	35.5	2.5
Middle temporal gyrus ([MTG], IC 62)	48.5	-60.5	10.5	Posterior cingulate cortex ([PCC], IC 71)	-5.5	-28.5	26.5
Cuneus (IC 15)	15.5	-91.5	22.5	Anterior cingulate cortex ([ACC], IC 17)	-9.5	46.5	-10.5
Right middle occipital gyrus ([R MOG], IC 12)	38.5	-73.5	6.5	Precuneus (IC 51)	-0.5	-48.5	49.5
Fusiform gyrus (IC 93)	29.5	-42.5	-12.5	Posterior cingulate cortex ([PCC], IC 94)	-2.5	54.5	31.5
Inferior occipital gyrus ([IOG], IC 20)	-36.5	-76.5	-4.5	**Cerebellar domain (CB)**			
Lingual gyrus ([LingualG], IC 8)	-8.5	-81.5	-4.5	Cerebellum ([CB], IC 13)	-30.5	-54.5	-42.5
Middle temporal gyrus ([MTG], IC 77)	-44.5	-57.5	-7.5	Cerebellum ([CB], IC 18)	-32.5	-79.5	-37.5
				Cerebellum ([CB], IC 4)	20.5	-48.5	-40.5
				Cerebellum ([CB], IC 7)	30.5	-63.5	-40.5

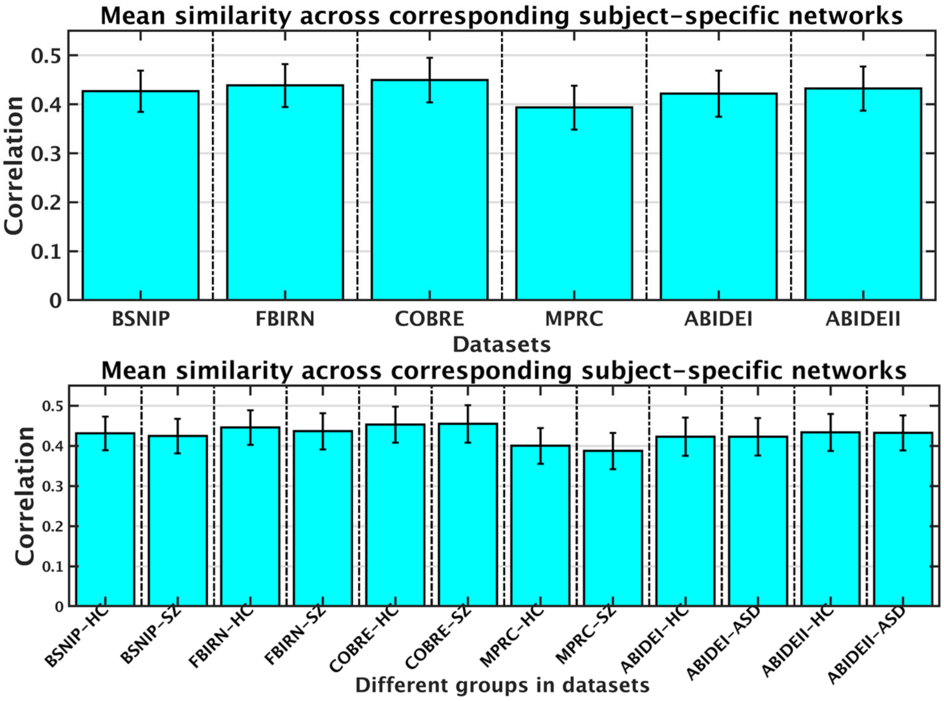

Fig. S2 Inter-subject similarity of functional networks is shown for all subjects in each dataset and the subjects in each group of the dataset, using error bars. For each dataset or each group in one dataset, the inter-subject similarity of 53 networks is shown using an errorbar. The result suggests that in general, the correlations across corresponding subject-specific networks were around 0.5, which accords to the multi-objective function in GIG-ICA.

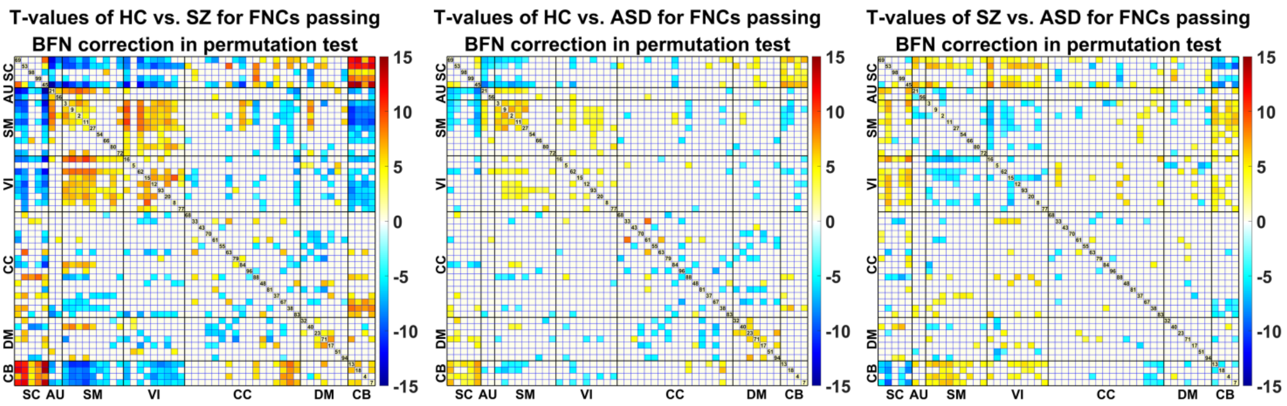

Fig. S3 Group differences that were obtained from a permutation test after Bonferroni (BFN) correction. Here, we show the T-values (HC vs. SZ, HC vs. ASD, and SZ vs. ASD using original groups) of FNCs that passed BFN correction according to the final p-values of the permutation test. It is seen that the group differences identified using the permutation test (Fig. S3) are quite consistent with that estimated using the direct two-sample t-test (Fig. 4), supporting that the group differences are reliable.

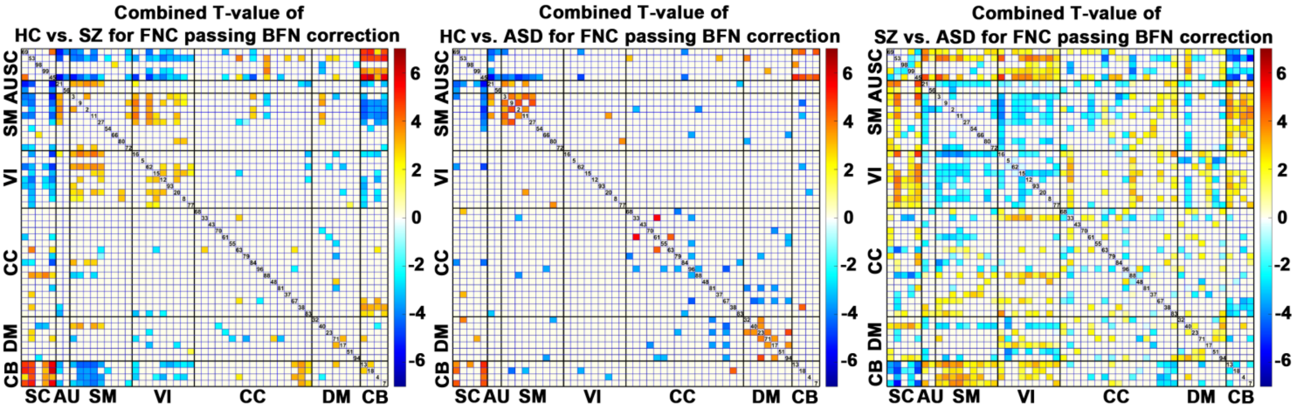

Fig. S4 The combined group differences (HC vs. SZ, HC vs. ASD, and SZ vs. ASD) that were obtained from a meta-analysis on separate datasets. The mean T-values across different comparisons are shown for the FNCs that passed Bonferroni (BFN) correction in terms of the combined p-values. Group differences in Fig. S4 show a similar pattern to that in Fig. 4, supporting that the identified overlap and uniqueness of brain abnormality were relatively reliable.

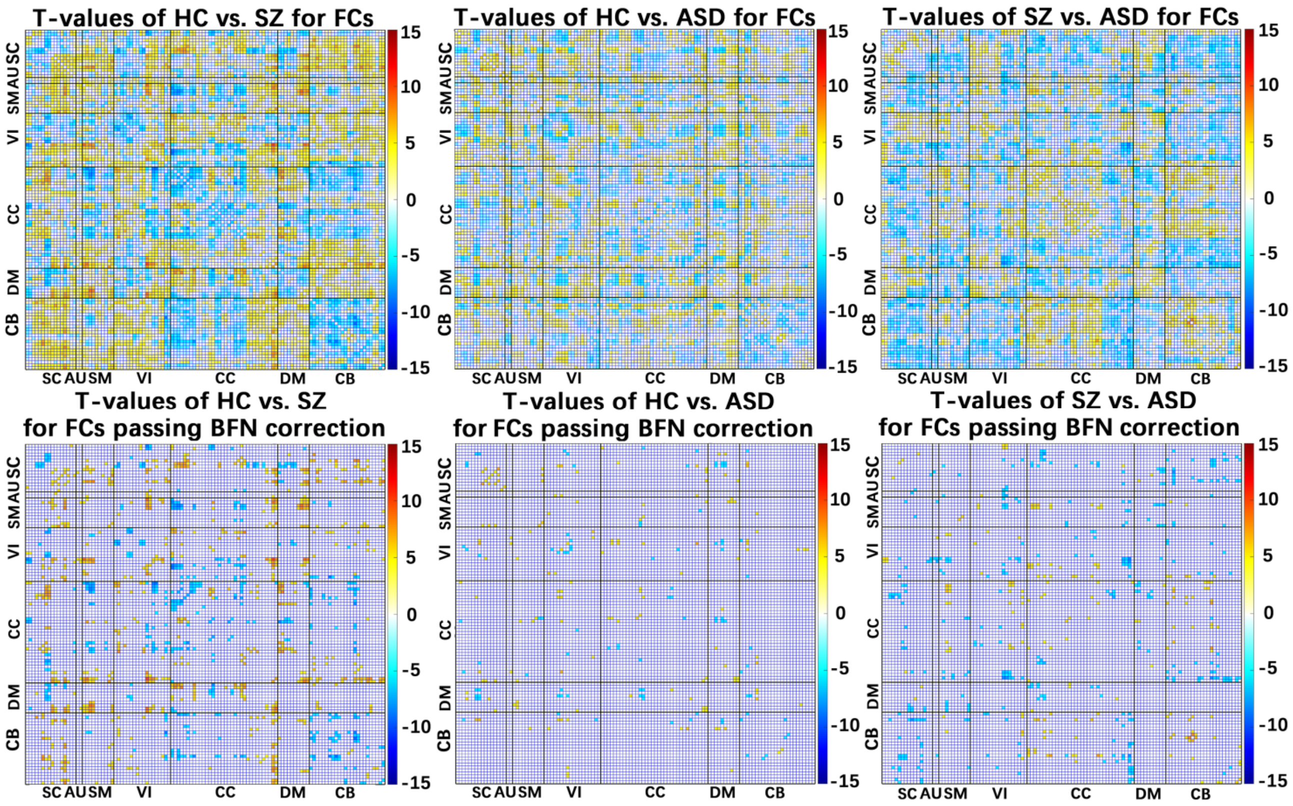

Fig. S5 Results of functional connectivity (FC) analysis using ROIs of Brainnetome atlas. Upper figures: the original T-value maps representing group differences in FCs revealed by two-sample t-tests for HC vs. SZ, HC vs. ASD, and SZ vs. ASD. Lower figures: the T-value maps of FCs passing Bonferroni (BFN) corrections.

Table S6. The brain regions that showed disorder-common or disorder-unique changes in more than 200 voxels, evaluated by statistical analyses with multiple comparison correction on gray matter volume. For each brain region, we included the relevant region name (in AAL atlas), the voxel number, and the mean p-value and mean T-value (across voxels) in HC vs. SZ, HC vs. ASD, and SZ vs. ASD (tested by two-sample t-test).

Disorder-common and disorder-unique changes	Brain region	Voxel number	Mean p-value across voxels in HC vs. SZ	Mean T-value across voxels in HC vs. SZ	Mean p-value across voxels in HC vs. ASD	Mean T-value across voxels in HC vs. ASD	Mean p-value across voxels in SZ vs. ASD	Mean T-value across voxels in SZ vs. ASD
Disorder-common decrease	Occipital-Mid-L	496	7.91e-10	7.23	1.73e-04	4.04	3.41e-01	-2.48
	Occipital-Mid-R	304	1.69e-10	7.21	1.79e-04	4.02	2.60e-01	-2.69
	Cerebelum-Crus2-L	588	1.29e-07	7.47	6.75e-05	4.92	4.10e-01	-2.36
	Cerebelum-Crus2-R	643	3.09e-08	8.26	7.74e-05	4.81	2.52e-01	-3.39
	Cerebelum-7b-R	209	1.54e-09	8.55	6.42e-05	4.65	1.53e-01	-3.93
Disorder-common increase	Cerebelum-8-R	549	3.15e-04	-4.18	3.24e-06	-5.44	1.00e+00	0.00
	Cerebelum-9-L	262	4.92e-05	-5.48	3.73e-05	-4.75	8.82e-01	0.40
	Cerebelum-9-R	309	5.09e-05	-5.27	7.07e-06	-5.35	9.81e-01	0.06
SZ-unique decrease (i.e. ASD-unique increase)	Frontal-Sup-R	203	1.55e-04	4.94	2.21e-04	-3.88	4.21e-09	-6.96
	Frontal-Mid-L	202	2.63e-04	4.40	3.28e-04	-3.70	1.50e-08	-6.33
	Frontal-Sup-Medial-R	214	1.75e-04	4.88	2.13e-04	-3.98	5.17e-09	-6.92
	Cingulum-Mid-L	203	3.74e-04	3.86	3.55e-04	-3.65	2.91e-08	-5.82
	Cingulum-Mid-R	328	3.11e-04	3.90	3.35e-04	-3.68	3.34e-08	-5.86
	Temporal-Sup-L	378	1.41e-04	4.98	3.28e-04	-3.73	1.02e-08	-6.81
	Temporal-Sup-R	690	2.45e-04	4.57	1.89e-04	-4.00	7.16e-09	-6.71
	Temporal-Pole-Sup-R	273	1.76e-04	4.86	2.01e-04	-4.00	7.20e-09	-6.82
	Temporal-Mid-L	822	1.98e-04	4.55	1.90e-04	-4.01	6.95e-09	-6.67
	Temporal-Mid-R	534	1.83e-04	4.72	1.87e-04	-4.05	5.86e-09	-6.85
	Temporal-Pole-Mid-R	249	1.68e-04	5.26	1.31e-04	-4.46	2.55e-09	-7.56
	Temporal-Inf-L	222	2.88e-04	4.47	2.34e-04	-3.96	9.96e-09	-6.50
	Temporal-Inf-R	224	2.40e-04	4.23	2.38e-04	-3.88	8.14e-09	-6.35

Table S7. The brain regions that showed disorder-common or disorder-unique changes in more than 200 voxels, evaluated by statistical analyses with multiple comparison correction on gray matter density. For each brain region, we included the relevant region name (in AAL atlas), the voxel number, and the mean p-value and mean T-value (across voxels) in HC vs. SZ, HC vs. ASD, and SZ vs. ASD (tested by two-sample t-test).

Disorder-common and disorder-unique changes	Brain region	Voxel number	Mean p-value across voxels in HC vs. SZ	Mean T-value across voxels in HC vs. SZ	Mean p-value across voxels in HC vs. ASD	Mean T-value across voxels in HC vs. ASD	Mean p-value across voxels in SZ vs. ASD	Mean T-value across voxels in SZ vs. ASD
Disorder-common decrease	Precentral-L	917	6.93e-10	8.62	1.44e-04	4.22	8.08e-02	-4.67
	Precentral-R	1003	1.22e-08	8.25	1.42e-04	4.23	2.45e-01	-3.92
	Frontal-Sup-L	747	1.01e-09	8.63	1.48e-04	4.19	6.71e-02	-4.65
	Frontal-Sup-R	688	4.00e-09	9.34	1.78e-04	4.01	5.68e-02	-5.44
	Frontal-Sup-Orb-L	525	9.52e-12	10.02	1.49e-04	4.17	2.29e-02	-5.86
	Frontal-Sup-Orb-R	758	4.61e-12	10.22	8.45e-05	4.46	3.31e-02	-5.73
	Frontal-Mid-L	1716	5.10e-11	10.12	1.34e-04	4.20	3.56e-02	-6.02
	Frontal-Mid-R	1590	5.56e-12	10.55	1.65e-04	4.10	2.52e-02	-6.47
	Frontal-Mid-Orb-L	830	1.97e-15	11.27	4.62e-05	4.83	3.86e-02	-6.43
	Frontal-Mid-Orb-R	1019	1.89e-11	11.00	3.94e-05	4.88	4.82e-02	-6.10
	Frontal-Inf-Oper-L	236	5.13e-12	9.89	1.93e-04	3.91	4.58e-05	-6.16
	Frontal-Inf-Tri-L	562	4.16e-13	10.57	1.73e-04	4.05	3.58e-03	-6.49
	Frontal-Inf-Tri-R	389	7.36e-16	10.93	1.80e-04	3.94	2.37e-06	-6.90
	Frontal-Inf-Orb-L	905	7.68e-11	10.75	1.09e-04	4.35	1.33e-02	-6.44
	Frontal-Inf-Orb-R	786	4.54e-11	11.06	1.08e-04	4.29	5.11e-03	-6.80
	Rolandic-Oper-L	230	5.97e-16	10.14	2.20e-04	3.91	1.23e-05	-6.40
	Frontal-Sup-Medial-L	374	1.08e-10	9.73	2.66e-04	3.77	3.48e-02	-5.89
	Frontal-Sup-Medial-R	295	5.65e-10	8.71	2.33e-04	3.83	2.38e-02	-5.11
	Rectus-R	345	1.14e-11	10.97	8.13e-05	4.54	2.61e-02	-6.30
	Insula-L	856	1.27e-12	12.71	2.35e-04	3.90	2.77e-06	-8.67
	Insula-R	633	8.29e-14	12.38	2.06e-04	4.02	2.46e-06	-8.36
	ParaHippocampal-L	339	1.73e-09	9.51	1.50e-04	4.11	6.50e-02	-5.54
	ParaHippocampal-R	409	1.22e-08	9.40	1.35e-04	4.42	1.52e-01	-4.70
	Calcarine-L	237	1.28e-10	8.28	2.00e-04	3.94	1.70e-02	-4.81
	Calcarine-R	284	3.59e-10	8.19	1.94e-04	3.97	6.71e-02	-4.60
	Lingual-R	285	6.06e-11	8.19	1.28e-04	4.37	1.12e-01	-4.22
	Occipital-Sup-L	296	5.00e-08	7.38	1.03e-04	4.45	3.14e-01	-2.74
	Occipital-Sup-R	368	4.59e-10	7.47	9.67e-05	4.31	2.37e-01	-3.11
	Occipital-Mid-L	1587	3.61e-14	9.31	4.28e-05	5.19	1.19e-01	-4.30
	Occipital-Mid-R	990	9.57e-14	9.32	3.99e-05	5.18	1.18e-01	-4.30
	Occipital-Inf-L	485	9.23e-15	9.65	4.28e-05	5.01	7.43e-02	-4.97

	Occipital-Inf-R	501	1.92e-14	9.87	4.92e-05	4.99	2.01e-02	-5.27
	Fusiform-L	366	4.36e-11	9.35	1.82e-04	4.08	3.02e-02	-5.58
	Fusiform-R	510	1.14e-09	9.74	1.28e-04	4.16	5.11e-02	-5.82
	Postcentral-L	1491	1.21e-08	9.04	1.17e-04	4.36	1.01e-01	-4.86
	Postcentral-R	840	6.24e-09	8.25	1.16e-04	4.44	2.10e-01	-3.75
	Parietal-Sup-L	440	4.70e-07	6.71	1.60e-04	4.35	5.48e-01	-2.04
	Parietal-Inf-L	682	1.17e-09	8.61	1.13e-04	4.52	1.54e-01	-4.35
	Parietal-Inf-R	369	2.76e-09	8.66	1.09e-04	4.42	1.41e-01	-4.36
	SupraMarginal-L	686	1.41e-14	10.05	7.28e-05	4.61	3.65e-02	-5.71
	SupraMarginal-R	862	6.45e-13	9.79	6.84e-05	4.59	6.04e-02	-5.39
	Angular-L	605	3.52e-13	10.02	5.34e-05	4.90	3.81e-02	-5.58
	Angular-R	840	9.11e-10	9.26	4.84e-05	5.08	1.86e-01	-4.29
	Caudate-L	557	2.34e-08	6.90	1.55e-04	3.97	4.44e-01	-2.46
	Caudate-R	413	4.98e-08	7.04	1.31e-04	4.12	3.32e-01	-2.81
	Thalamus-L	529	2.29e-10	8.91	1.03e-04	4.37	5.87e-02	-4.81
	Thalamus-R	714	8.66e-10	8.61	5.67e-05	4.88	2.10e-01	-3.84
	Temporal-Sup-L	347	7.98e-12	9.27	1.50e-04	4.23	8.08e-02	-5.16
	Temporal-Sup-R	411	2.32e-15	9.12	1.82e-04	3.98	2.49e-03	-5.22
	Temporal-Mid-L	1419	1.38e-12	9.95	1.04e-04	4.50	4.66e-02	-5.58
	Temporal-Mid-R	1415	5.81e-15	10.10	9.99e-05	4.41	1.70e-02	-5.86
	Temporal-Pole-Mid-L	215	3.83e-15	10.24	2.21e-04	3.96	1.20e-05	-6.46
	Temporal-Pole-Mid-R	559	3.06e-13	11.06	1.30e-04	4.17	1.79e-03	-6.97
	Temporal-Inf-L	1349	8.25e-10	9.09	1.36e-04	4.19	8.02e-02	-5.02
	Temporal-Inf-R	1681	1.84e-10	9.79	1.04e-04	4.45	4.77e-02	-5.45
	Cerebelum-Crus1-L	1023	1.53e-08	7.56	9.43e-05	4.81	3.88e-01	-2.71
	Cerebelum-Crus1-R	775	3.98e-10	9.36	8.31e-05	4.81	1.23e-01	-4.74
	Cerebelum-Crus2-L	1610	5.46e-09	8.89	3.50e-05	5.82	2.64e-01	-3.49
	Cerebelum-Crus2-R	1846	3.97e-08	9.14	3.44e-05	5.69	2.72e-01	-3.70
	Cerebelum-7b-L	431	5.45e-08	8.61	2.70e-05	5.63	3.67e-01	-3.20
	Cerebelum-7b-R	548	1.20e-08	9.65	2.86e-05	5.79	2.23e-01	-4.14
	Cerebelum-8-L	1442	5.64e-09	7.80	7.96e-05	4.40	2.11e-01	-3.25
	Cerebelum-8-R	1755	1.04e-08	8.35	5.89e-05	4.68	3.05e-01	-3.42

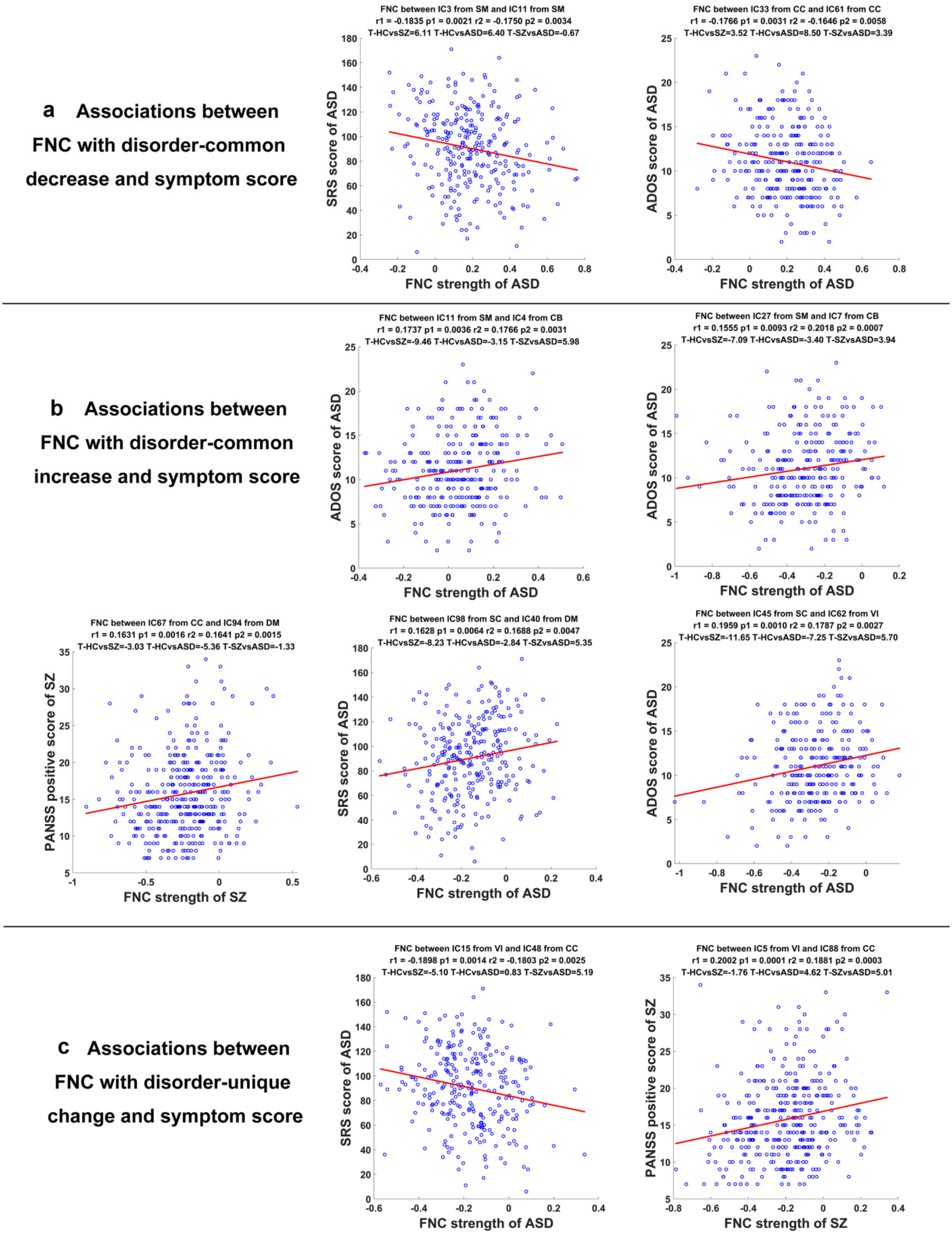

Fig. S6 Associations between the FNC strengths and symptom scores of SZ and ASD, measured by Pearson correlation and Spearman rank correlation. a, b, and c include the correlations for the FNCs with

disorder-common decrease, the FNCs with disorder-common increase, and the FNCs with disorder-unique changes, respectively. In the title of each subfigure, we show Pearson correlation (r1 and p1) and Spearman rank correlation (r2 and p2) for reflecting the association and we also show T-values obtained from two-sample t-tests on any two groups for reflecting the group difference.

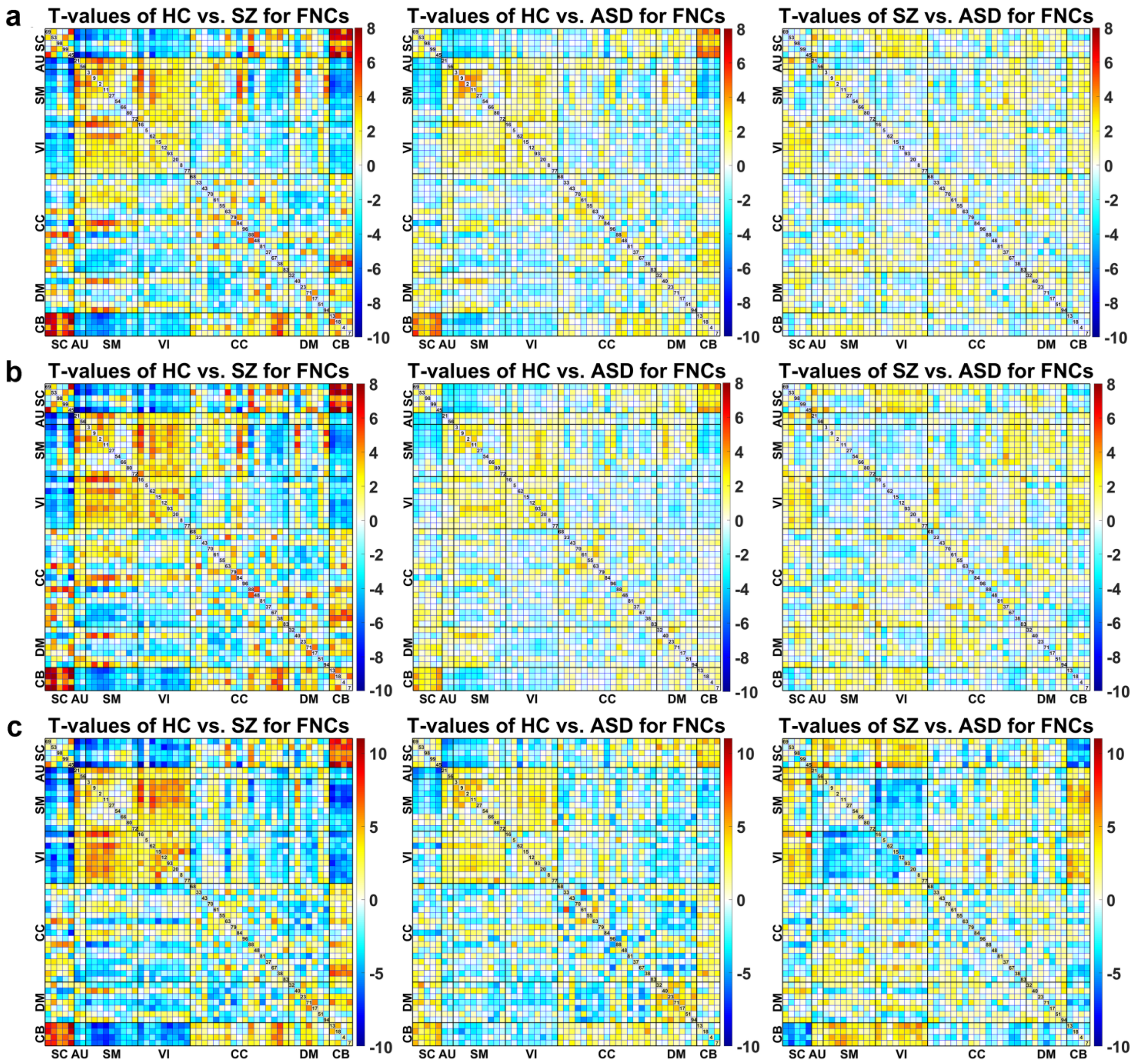

Fig. S7 FNC group differences using the subjects with matched age and the subjects with no motion difference. **a** and **b** show the group differences obtained using the sample set 1 and 2 (each of them included age-matched subjects but they had different age ranges), respectively. **c** shows the group differences obtained using the subjects with no motion difference. T-values obtained using two-sample t-tests on any pair of groups are displayed to compare with the results (Fig. 4) estimated using all subjects.

Table S8. Data from separate datasets used for identifying group differences in FNC

Investigation of group difference	Datasets used
**HC vs. SZ**	BSNIP; FBIRN; COBRE; MPRC
**HC vs. ASD**	ABIDEI; ABIDEII
**SZ vs.ASD**	BSNIP and ABIDEI; BSNIP and ABIDEII; FBIRN and ABIDEI; FBIRN and ABIDEII; COBRE and ABIDEI; COBRE and ABIDEII; MPRC and ABIDEI; MPRC and ABIDEII

Table S9. Information of two sample sets, each of which included age-matched HC, SZ, and ASD groups. The two sample sets had slightly different age ranges.

			HC	SZ	ASD
**Sample set 1**	**Subject number**		442	222	130
	**Age**	**Range**	[21, 35]	[21, 35]	[21, 36]
		**Mean**	26.61	27.09	26.91
		**Std**	3.88	4.17	4.32
	**p-value of age among the three groups, tested by ANOVA**		0.0797		
**Sample set 2**	**Subject number**		461	248	104
	**Age**	**Range**	[23, 42]	[23, 42]	[23, 42]
		**Mean**	30.50	31.09	29.60
		**Std**	5.67	5.74	5.49
	**p-value of age among the three groups, tested by ANOVA**		0.0731		

Note: Std denotes standard deviation.

Table S10. Information of the selected subjects with no motion differences across the HC, SZ, and ASD groups.

	**HC**	**SZ**	**ASD**
**Subject number**	838	212	513
**Motion transitions: mean (std)**	0.2431 (0.1327)	0.2578 (0.1395)	0.2485 (0.1443)
**Motion rotations: mean (std)**	0.2522 (0.1317)	0.2464 (0.1497)	0.2624 (0.1482)
**p-value of motion transitions, tested by ANOVA**	0.3616		
**p-value of motion rotations, tested by ANOVA**	0.2761		
