# Supplementary material for: Evidence of shared and distinct functional and structural brain signatures in schizophrenia and autism spectrum disorder
Source: Commun Biol. 2021 Sep 14;4:1073. doi: 10.1038/s42003-021-02592-2 (PMC8440519; doi:10.1038/s42003-021-02592-2)
Supplement: Supplementary file 3 — Reporting Summary [file 42003_2021_2592_MOESM3_ESM.pdf]

nature research

Corresponding author(s): Yuhui Du

Last updated by author(s): Jul 23, 2021

Reporting Summary

Nature Research wishes to improve the reproducibility of the work that we publish. This form provides structure for consistency and transparency in reporting. For further information on Nature Research policies, seeAuthors & Referees and theEditorial Policy Checklist .

Statistics

For all statistical analyses, confirm that the following items are present in the figure legend, table legend, main text, or Methods section.

n/a	Confirmed	
☐	☒	The exact sample size (n) for each experimental group/condition, given as a discrete number and unit of measurement
☐	☒	A statement on whether measurements were taken from distinct samples or whether the same sample was measured repeatedly
☐	☒	The statistical test(s) used AND whether they are one- or two-sided<br>*Only common tests should be described solely by name; describe more complex techniques in the Methods section.*
☐	☒	A description of all covariates tested
☐	☒	A description of any assumptions or corrections, such as tests of normality and adjustment for multiple comparisons
☐	☒	A full description of the statistical parameters including central tendency (e.g. means) or other basic estimates (e.g. regression coefficient) AND variation (e.g. standard deviation) or associated estimates of uncertainty (e.g. confidence intervals)
☐	☒	For null hypothesis testing, the test statistic (e.g. F, t, r) with confidence intervals, effect sizes, degrees of freedom and P value noted<br>*Give P values as exact values whenever suitable.*
☒	☐	For Bayesian analysis, information on the choice of priors and Markov chain Monte Carlo settings
☐	☒	For hierarchical and complex designs, identification of the appropriate level for tests and full reporting of outcomes
☐	☒	Estimates of effect sizes (e.g. Cohen's d, Pearson's r), indicating how they were calculated

Our web collection on statistics for biologists contains articles on many of the points above.

Software and code

Policy information about availability of computer code

Data collection	The data that we used are open sources, including Bipolar--Schizophrenia Network for Intermediate Phenotypes (BSNIP-1), Function Biomedical Informatics Research Network (FBIRN), Centers of Biomedical Research Excellence (COBRE), Maryland Psychiatric Research Center (MPRC), Autism Brain Imaging Data Exchange I (ABIDEI) and ABIDEII.
Data analysis	We used SPM12 for the preprocessing of data.

For manuscripts utilizing custom algorithms or software that are central to the research but not yet described in published literature, software must be made available to editors/reviewers. We strongly encourage code deposition in a community repository (e.g. GitHub). See the Nature Research guidelines for submitting code & software for further information.

Data

Policy information about availability of data

All manuscripts must include a data availability statement. This statement should provide the following information, where applicable:

- Accession codes, unique identifiers, or web links for publicly available datasets
- A list of figures that have associated raw data
- A description of any restrictions on data availability

The data that support the findings of this study are available from the corresponding author upon reasonable request. The functional network templates used in this paper are available online (www.yuhuidu.com and http://trendscenter.org/software).

Field-specific reporting

Please select the one below that is the best fit for your research. If you are not sure, read the appropriate sections before making your selection.

Life sciences study design

All studies must disclose on these points even when the disclosure is negative.

Sample size	After preprocessing and quality control, we estimated brain functional measures from resting functional magnetic resonance imaging (fMRI) data of 2980 subjects (1665 HCs, 537 SZs, and 778 ASDs), gray matter volume from structural MRI (sMRI) data of 3148 subjects (1661 HCs, 517 SZs, and 970 ASDs), and gray matter density from sMRI data of 3374 subjects (1789 HCs, 555 SZs, and 1030 ASDs).
Data exclusions	In this study, we set out some criteria for fMRI and sMRI respectively to ensure that we used high-quality data and brain masks for analysis. Please find the details in the supplementary materials.
Replication	The findings are replicated using results from separate studies, using results from age-matched subjects, using results from subjects with no motion difference, and using results from classification.
Randomization	We carefully regressed out the influences of age, gender and site effects for each subject from the estimated functional and structural measures. The regression procedure included three steps. First, the age, gender, site factor, and the interaction between any two factors were regressed out for all subjects in each study (BSNIP, FBIRN, COBRE, MPRC, ABIDEI and ABIDEII). Then, the study effects were estimated using healthy controls' measures from the six studies. Finally, we further regressed out the study effect from each subject's measures.
Blinding	The investigators were blinded to the samples used.

Reporting for specific materials, systems and methods

We require information from authors about some types of materials, experimental systems and methods used in many studies. Here, indicate whether each material, system or method listed is relevant to your study. If you are not sure if a list item applies to your research, read the appropriate section before selecting a response.

Materials & experimental systems

n/a	Involved in the study
☒	☐ Antibodies
☒	☐ Eukaryotic cell lines
☒	☐ Palaeontology
☒	☐ Animals and other organisms
☐	☒ Human research participants
☐	☒ Clinical data

Methods

n/a	Involved in the study
☒	☐ ChIP-seq
☒	☐ Flow cytometry
☐	☒ MRI-based neuroimaging

Human research participants

Policy information about studies involving human research participants

Population characteristics	In our paper, there are multi-site datasets from six studies included for analyses. The demographic information for the used data is shown in Table S1-S4. We have regressed out the effects of age, gender and site before statistical analyses.
Recruitment	FMRI and sMRI data were collected from six multi-site studies. Most of these studies are open sources.
Ethics oversight	Bipolar--Schizophrenia Network for Intermediate Phenotypes (BSNIP-1), Function Biomedical Informatics Research Network (FBIRN), Centers of Biomedical Research Excellence (COBRE), Maryland Psychiatric Research Center (MPRC), Autism Brain Imaging Data Exchange I (ABIDEI) and ABIDEII.

Note that full information on the approval of the study protocol must also be provided in the manuscript.

Clinical data

Policy information about clinical studies

All manuscripts should comply with the ICMJEguidelines for publication of clinical research and a completedCONSORT checklist must be included with all submissions.

Clinical trial registration	The data we used are open sources. So, we do not have the information.
Study protocol	Bipolar--Schizophrenia Network for Intermediate Phenotypes (BSNIP-1), Function Biomedical Informatics Research Network (FBIRN), Centers of Biomedical Research Excellence (COBRE), Maryland Psychiatric Research Center (MPRC), Autism Brain Imaging Data Exchange I (ABIDEI) and ABIDEII
Data collection	Different parameters were used for different sites as we included multiple sites. So, we cannot list them here.
Outcomes	Not applicable.

Magnetic resonance imaging

Experimental design

Design type	Resting data
Design specifications	Too many datasets are included in our paper. The multi-site data have different designs. So, we cannot list them here.
Behavioral performance measures	We included the symptom scores (not the behavioral measures).

Acquisition

Imaging type(s)	functional and structural MRI
Field strength	Different parameters were used for different sites while we included multiple sites. So, we cannot list them here.
Sequence & imaging parameters	Different parameters were used for different sites while we included multiple sites. So, we cannot list them here.
Area of acquisition	Different parameters were used for different sites while we included multiple sites. So, we cannot list them here.

Diffusion MRI ☐ Used ☒ Not used

Preprocessing

Preprocessing software

We preprocessed the fMRI and sMRI data using the latest statistical parametric mapping toolbox (SPM12).
For fMRI data, we removed the first six time points and then performed the rigid body motion correction to correct the subject head motion, followed by the slice-timing correction to account for timing difference in slice acquisition. The fMRI data were subsequently warped into the standard Montreal Neurological Institute (MNI) space using an echo planar imaging (EPI) template and were then resampled to 3 × 3 ×3 mm3 isotropic voxels. The resampled fMRI images were further smoothed using a Gaussian kernel with a full width at half maximum (FWHM) = 6 mm. The smoothed fMRI data were used for ICA. For the ROI-based method, the smoothed data were further detrended and band-pass filtered [0.01Hz-0.15Hz], followed by regressing out nuisance covariates including six head motion parameters, white matter signal, cerebrospinal fluid signal, and global mean signal.
For sMRI data, the T1-weighted images were first segmented into gray matter, white matter, and cerebrospinal fluid by using the standard unified segmentation model (Ashburner and Friston 2005). The Diffeomorphic Anatomical Registration Through Exponentiated Lie Algebra (DARTEL) algorithm was employed to create a group template for spatial normalization of the segmented images of each subject. Then, the flow fields generated by DARTEL were used to estimate individual-subject images. After that, individual-subject gray matter images were spatially normalized to the MNI space, modulated or unmodulated, resliced (1.0-mm isotropic voxels), and smoothed (6-mm full-width at half maximum Gaussian kernel). Finally, the obtained gray matter volume (modulated data) and density (unmodulated data) can be used for voxel-based morphometry (VBM).

Normalization	Please see the above descriptions for both fMRI and sMRI.
Normalization template	Montreal Neurological Institute (MNI) space
Noise and artifact removal	For fMRI, we regressed out nuisance covariates including six head motion parameters, white matter signal, cerebrospinal fluid signal, and global mean signal.
Volume censoring	*Define your software and/or method and criteria for volume censoring, and state the extent of such censoring.*

Statistical modeling & inference

Model type and settings	Univariate analysis was used.
Effect(s) tested	ANOVA was used before two-tailed two-sample t-tests.

Specify type of analysis: ☐ Whole brain ☐ ROI-based ☒ Both

Anatomical location(s): Both independent component analysis and atlas were used for functional connectivity computation.

Statistic type for inference (See Eklund et al. 2016)	voxel-wise statistical analysis for sMRI
Correction	Bonferroni correction

Models & analysis

n/a	Involved in the study
☐	☒ Functional and/or effective connectivity
☒	☐ Graph analysis
☐	☒ Multivariate modeling or predictive analysis

Functional and/or effective connectivity	Pearson correlation was used for measuring functional dependence.
Multivariate modeling and predictive analysis	Support vector machine was used for classification.
